# Coronary Event Risk Test (CERT) as a Risk Predictor for the 10-Year Clinical Outcome of Patients with Peripheral Artery Disease

**DOI:** 10.3390/jcm12196151

**Published:** 2023-09-23

**Authors:** Andreas Leiherer, Axel Muendlein, Christoph H. Saely, Kathrin Geiger, Eva-Maria Brandtner, Christine Heinzle, Stella Gaenger, Sylvia Mink, Reijo Laaksonen, Peter Fraunberger, Heinz Drexel

**Affiliations:** 1 Vorarlberg Institute for Vascular Investigation and Treatment (VIVIT), Academic Teaching Hospital Feldkirch, Carinagasse 47, A-6800 Feldkirch, Austria; axel.muendlein@vivit.at (A.M.); kathrin.geiger@vivit.at (K.G.); lilli.brandtner@vivit.at (E.-M.B.); stella.gaenger@vivit.at (S.G.); heinz.drexel@vivit.at (H.D.); 2 Private University of the Principality of Liechtenstein, FL-9495 Triesen, Liechtenstein; smink@mzl.at (S.M.); pfraunberger@mzl.at (P.F.); 3 Medical Central Laboratories, A-6800 Feldkirch, Austria; 4 Department of Internal Medicine III, Academic Teaching Hospital Feldkirch, A-6800 Feldkirch, Austria; 5 Finnish Cardiovascular Research Center, University of Tampere, FI-33014 Tampere, Finland; reijo.laaksonen@zora.fi; 6 Zora Biosciences, FI-02150 Espoo, Finland; 7 Vorarlberger Landeskrankenhausbetriebsgesellschaft, Academic Teaching Hospital Feldkirch, A-6800 Feldkirch, Austria; 8 Drexel University College of Medicine, Philadelphia, PA 19129, USA

**Keywords:** peripheral artery disease, biomarker, ceramides, CERT, mortality, MACE, risk factors, statin

## Abstract

(1) Background: Ceramides are a new kind of lipid biomarker and have already been demonstrated to be valuable risk predictors in coronary patients. Patients with peripheral artery disease (PAD) are a population with a worse prognosis and higher mortality risk compared to coronary artery disease (CAD) patients. However, the value of ceramides for risk prediction in PAD patients is still vague, as addressed in the present study. (2)Methods: This observational study included 379 PAD patients. The primary endpoint was all-cause mortality at 10 years of follow-up. A set of ceramides was measured by LC-MS/MS and combined according to the Coronary Event Risk Test (CERT) score, which categorizes patients into one of four risk groups (low risk, moderate risk, high risk, very high risk). (3) Results: Kaplan–Meier survival curves revealed that the overall survival of patients decreased with the increasing risk predicted by the four CERT categories, advancing from low risk to very high risk. Cox regression analysis demonstrated that each one-category increase resulted in a 35% rise in overall mortality risk (HR = 1.35 [1.16–1.58]). Multivariable adjustment, including, among others, age, LDL-cholesterol, type 2 diabetes, and statin treatment before the baseline, did not abrogate this significant association (HR = 1.22 [1.04–1.43]). Moreover, we found that the beneficial effect of statin treatment is significantly stronger in patients with a higher risk, according to CERT. (4) Conclusions: We conclude that the ceramide-based risk score CERT is a strong predictor of the 10-year mortality risk in patients with PAD.

## 1. Introduction

Atherosclerosis is a systemic disorder characterized by the accumulation of lipid-rich plaques within the arterial walls. This pathological process occurs throughout the body, causing the progressive narrowing and stiffening of arteries. One specific manifestation of atherosclerosis is peripheral artery disease (PAD). It primarily affects the arteries outside the heart and brain, most commonly in the lower extremities [1]. PAD is a chronic and progressive disease; it leads to reduced blood flow to the legs and feet, resulting in claudication, ischemic ulcers, and tissue necrosis, and, if left untreated, it significantly contributes to morbidity and mortality, leading to considerable rates of disability and death, which are attributed to stroke and myocardial infarction [2]. Compared to coronary artery disease (CAD), patients with PAD are at a significantly higher risk of cardiovascular events [1,3,4], and their long-term prognosis is even less favorable [5]. 

Recent studies have shown that lipid-lowering therapy by statins significantly increases the overall survival of PAD patients [6], but many patients with PAD do not experience any symptoms at an early disease stage [7]. Indeed, PAD tends to be frequently underdiagnosed and undertreated despite its severity as a systemic condition. Consequently, there is a pressing need for the improved recognition and therapy of PAD to mitigate its detrimental impact on public health.

A detailed comparison between PAD and CAD has recently demonstrated that ceramide-based lipid profiles are able to discriminate between PAD and CAD [8]. Ceramides are sphingolipids and have recently been described as novel lipid biomarkers that are involved in several signaling processes, exerting a wide range of biological functions [9]. They modulate cellular and whole-body metabolism and are associated with a number of diseases [10]. They are also found in coronary plaque and are involved in their formation [11]; however, the family of ceramides is large, and its members are distributed differently among lipoprotein particles [12]. From a biological and clinical point of view, they can be divided into harmful species, indicating an increased cardiovascular risk and more neutral or benign forms [13,14,15]. Previous studies have shown that the prognostic test score named the Coronary Event Risk Test (CERT), which combines risk-associated and benign ceramides is superior to traditional cardiovascular risk markers such as LDL-cholesterol (LDL-C) in predicting cardiovascular risk [16,17].

However, in PAD patients, the prognosis of clinical outcomes for individual patients remains challenging, and for successful treatment, knowledge about the overall risk of PAD patients is important. As of yet, there is no single biomarker that is considered ideal for the diagnosis and management of PAD, and the commonly used ankle–brachial index (ABI) has its own set of limitations [18]. 

Therefore, the aim of this observational study is to elucidate the extent to which the ceramide-based score CERT can predict the clinical outcomes of patients with PAD. Additionally, we aim to investigate any potential interaction between CERT and the effects of statin treatment. To achieve this, we conducted an analysis of all-cause mortality risk and cardiovascular outcomes within a meticulously characterized cohort of patients with sonographically confirmed PAD, taking into consideration the patients’ statin treatment status prior to enrollment.

## 2. Methods

### 2.1. Study Subjects

Between October 2006 and December 2010, we consecutively enrolled 379 Caucasian patients who underwent routine duplex sonography for the evaluation of suspected or established PAD at the Academic Teaching Hospital Feldkirch and in whom PAD was sonographically verified. The clinical severity of PAD was classified according to the ankle–brachial index (ABI) and Rutherford stages [19]. Patients with acute coronary syndrome, secondary inflammation, wounds, or missing liquid chromatography-tandem mass spectrometry (LC-MS/MS) data were not included. The follow-up time was extended to 10 years and ended in November 2020. During the follow-up period, we recorded all-cause deaths (primary endpoint) as well as major cardiovascular events (MACE) and cardiovascular deaths (secondary endpoints). MACEs were defined as a composite of three factors: cardiovascular mortality, nonfatal myocardial infarction, and nonfatal stroke. Cardiovascular mortality was specifically defined as death resulting from myocardial infarction, sudden cardiac death, congestive heart failure due to CAD, or a fatal ischemic stroke. Myocardial infarction was diagnosed in the presence of at least two of three criteria: (i) standard electrocardiographic criteria, (ii) ischaemic cardiac pain, and (iii) creatinine kinase isoenzyme MB activity of at least twice the upper limit that is normal. A stroke was defined as a neurological deficit lasting longer than 48 h with a confirmative computer tomography or magnetic resonance image. The collection of data on both the date and cause of death was performed annually by utilizing a national registry (Statistik Austria, Vienna, Austria), hospital registries, and telephone contacts. Additionally, standardized interviews were conducted every two years to obtain information on nonfatal events. 

### 2.2. Clinical and Laboratory Analyses

Venous blood samples were collected after an overnight fast of 12 h before sonography was performed, and laboratory measurements were performed using fresh serum samples. Low-density lipoprotein cholesterol (LDL-C) was measured using enzymatic hydrolysis and precipitation techniques on a Hitachi-Analyzer 717 or 911 (QuantolipLDL, QuantolipHDL; Roche, Basel, Switzerland). Ceramides (Cer(d18:1/16:0), Cer(d18:1/18:0), Cer(d18:1/24:1), and Cer(d18:1/24:0)) were determined using (LC-MS/MS). For LC-MS/MS analysis, lipids were extracted from serum or plasma samples, as described previously [17]. In short, 10 µL of the sample was combined with 590 µL isopropanol/ethylacetate (8:2), containing the corresponding isotopically labeled standard for each ceramide. Samples were then mixed by aspirating them three times, followed by 10 min of centrifugation at 3000 g. Supernatants were transferred to an Eppendorf Twintec PCR plate and sealed with heat-sealing foil (Hamburg, Germany) prior to analysis with LC-MS/MS. Ceramide concentrations were calculated using standard straight calibration based on an internal standard concentration. The analyzed lipids and ions used in this study are presented in Supplementary Table S1. LC-MS/MS analysis was conducted on a Sciex MSTrap 5500 mass spectrometer coupled with the Shimadzu nexera 2 UHPLC system. Electrospray ionization in the positive ion mode was used with multiple reaction monitoring. Instrument and data acquisition were controlled using Analyst^®^ (version 1.6). The following settings were applied to all compounds in the analysis: curtain gas, 35; ion spray voltage, 5000 V; temperature, 300 °C; gas 1 and gas 2, 50; declustering potential, 30; entrance potential, 10; and collision exit potential, 20. Collision energy was set separately for each lipid (Supplementary Table S1). Chromatographic separation was performed on an Acquity BEH C18 2.1 × 50 mm id.1.7 µm column. The temperature was set to 60 °C. Mobile phases consisted of (A) 10 mM ammonium acetate with 0.1% formic acid and (B) 10 mM ammonium acetate in acetonitrile:2-propanol (4:3, *v*/*v*) with 0.1% formic acid. The injection volume was 3 µL, and the flow rate was 500 µL/min. The following gradient was applied: A/B (22/78%) from 0 to 1.5 min, then B to 85% at 2 min and to 100% at 2.5 min. B was held at 100% from 2.5 min to 4.0 min and then dropped to 78% at 4.1 min before it was held until 4.6 min. The selection of ceramides and their combined use in scores (CERT) has been described previously by others [17]. In short, the CERT score consists of three single ceramides and three ceramide/ceramide ratios. It assigns patients to a 13-step score ranging from 0 to 12 and, more generally, to one out of four risk categories: category 1 = low risk (score 0–2), category 2 = medium risk (score 3–6), category 3 = high risk (score 7–9), and category 4 = very high risk (score 10–12)). A more detailed description is provided elsewhere [17]. 

Type 2 diabetes mellitus (T2DM) was diagnosed according to the WHO definition [20]. Hypertension was diagnosed by adhering to NCEP-ATPIII criteria for high blood pressure or by identifying the use of antihypertensive treatment. Renal function was assessed based on the estimated glomerular filtration rate (eGFR) and classified as chronic kidney disease (CKD) in patients with eGFR less than 60 mL/min/1.73 m^2^, according to the guidelines of the Kidney Disease Outcomes Quality Initiative (KDOQI) [21]. Body mass index (BMI) was calculated as body weight (kg)/height^2^ (m). 

### 2.3. Statistical Analyses

Normal distribution was checked using the Kolmogorov–Smirnov test. Non-normally distributed variables were described using the median and interquartile range (IQR). Differences were tested for statistical significance with Chi-squared tests for categorical variables and with the Welch test for two continuous variables. In case of more than two continuous variables, we used the non-parametric Jonckheere–Terpstra test. Correlations was tested using the Pearson test. Survival curves were generated using the Kaplan–Meier method and compared by log-rank, Mantel–Cox tests. Hazard ratios (HRs) and 95% confidence intervals (CIs) of the HRs were derived from univariable and multivariable Cox proportional hazards models. Significance was defined as a two-tailed *p*-value < 0.05. No imputation was applied, and all data were analyzed using complete-case analysis. All statistical analyses were performed with SPSS 28.0 for Windows (IBM Corp.) and R statistical software v. 3.5.1 (http://www.r-project.org).

### 2.4. Ethics Approval and Consent Statements

The present study conforms to the ethical guidelines of the 1975 Declaration of Helsinki and was approved by the Ethics Committee of Vorarlberg, Austria (EK-2-22013/0008). Written informed consent was given by all participants. The authors affirm that all participants provided informed consent for the publication of anonymized data. 

## 3. Results

### 3.1. Follow-Up

In total, we enrolled 379 PAD patients. The median age of our patients at the baseline was 67 years. They were followed for a maximum of ten years, resulting in 2825 patient years. The median follow-up time of our patients was 9.0 years, with an interquartile range of 5.3 to 10.0 years. Follow-up data were available for 373 patients, and 6 patients were lost before the first follow-up interview. Out of these 373 patients, 159 (42%) died from any cause during follow-up, 138 (36%) suffered a MACE, and 66 (17%) succumbed to cardiovascular death. Forty-two (11%) have not been reached during the final 10-year follow-up interview; thus, partial and complete follow-up rates were 98% and 87%, respectively.

### 3.2. CERT Categories Predict Overall Mortality

To evaluate the power of the CERT score in PAD patients, we stratified all patients according to the 4 risk categories of the CERT score (low risk, moderate risk, high risk, and very high risk), which is only based on ceramide measures and does not use any demographic or anthropometric data. The baseline characteristics of all patients and the 4 subgroups are shown in Table 1. Therefore, there was no significant difference between the 4 groups regarding LDL-C levels (category 1: 96 mg/dL, category 2: 105 mg/dL, category 3: 111 mg/dL, and category 4: 92 mg/dL; *p* = 0.716). In addition, there was also no significant difference regarding patients’ sex, BMI, creatinine, hypertension prevalence, statin use, or smoking status. By contrast, age and the prevalence of T2DM and CKD increased with increasing CERT categories ranging from 1 (low risk) to 4 (very high risk). The severity of PAD was also significantly linked with CERT in terms of ABI and Rutherford stages.

**Figure 1:**
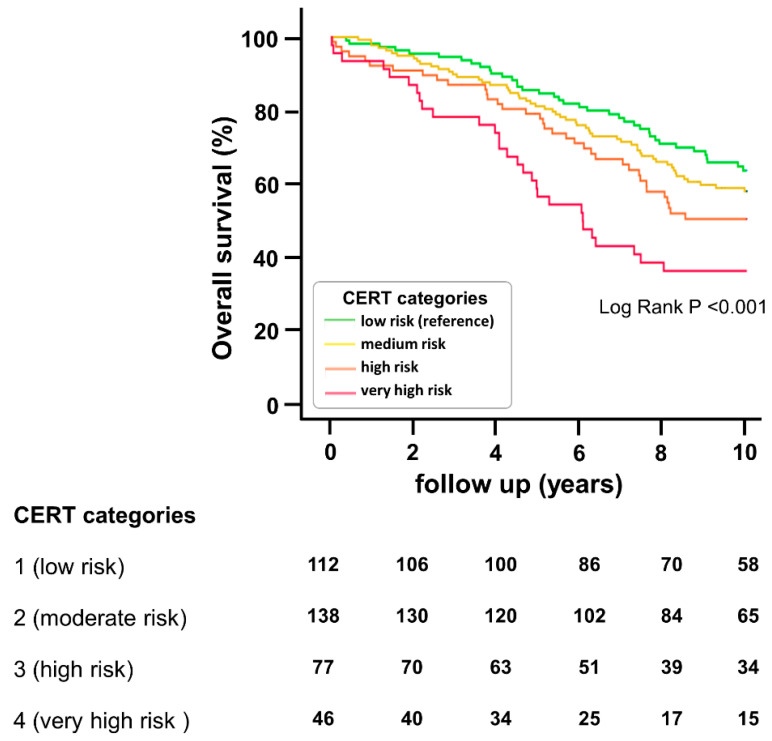
Overall mortality in PAD patients with respect to CERT risk groups. The plot depicts the Kaplan–Meier estimates for the cumulative probabilities of overall mortality according to the four risk categories of CERT. The number of patients at risk is given for each risk group every two years.

We then analyzed patients’ survival during the 10 years of follow-up. Kaplan–Meier survival curves are depicted in Figure 1. They demonstrate that overall survival significantly decreased with increasing CERT categories from low risk to very high risk. Comparable results are seen in the case of the secondary endpoints cardiovascular mortality and MACE. Respective survival curves are given in Supplementary Figure S1. Applying Cox regression, we found that each one-category increase in CERT (ranging from 1 to 4) resulted in a 35% rise in the overall mortality risk (HR = 1.351 [1.157–1.578], *p* ≤ 0.001). Alternatively, when applying the more detailed 13-step scoring scale of CERT (ranging from 0 to 12), every 1-step increase in the risk scale was associated with a 9% rise in the overall mortality risk (HR = 1.089 [1.040–1.114], *p* ≤ 0.001. The HRs of every single ceramide are summarized in Supplementary Table S2).

To account for possible confounding effects, we built several adjustment models (Table 2). We found that in a multivariate model including age, sex, BMI, LDL-C, the status of hypertension, CKD, T2DM, smoking, and statin treatment, CERT was still a significant predictor of overall mortality when applying the 4-category scale (HR = 1.215 [1.035–1.427], *p* = 0.017) and the 13-step scale (HR = 1.055 [1.007–1.106], *p* = 0.024), respectively. Apart from CERT, only age (HR = 1.070 [1.047–1.093], *p* < 0.001) and sex (HR = 2.343 [1.501–3.659], *p* < 0.001) were significant risk predictors in this model, whereas all other variables, including LDL-C, failed. Further models describing the association between CERT and the primary endpoint overall mortality, as well as between CERT and the secondary endpoints cardiovascular mortality and MACE, demonstrate comparable results (Table 2).

In contrast to CERT, ABI was no predictor of overall mortality (HR = 0.968 [0.800–1.171], *p* = 0.736), and there was no correlation between ABI and CERT (r = −0.062, *p* = 0.235).

### 3.3. Statin Effect Is Stronger in Higher-Risk Groups

Furthermore, we analyzed the effect of statin therapy on the outcome of patients. Seventy percent of our PAD patients were already undertaking statin therapy before enrollment. Comparing the pre-baseline-treated to the untreated patient subgroup using the one-sided Welch test, we found that mean LDL-C was significantly lower in the treated group than in the untreated one (99 ± 34 mg/dL vs. 123 ± 43 mg/dL, *p* < 0.001). Regarding the 4-category scale CERT in treated vs. untreated patients, there was no significant difference (2.1 ± 1.0 vs. 2.2 ± 0.9, *p* = 0.113). Similarly, regarding the distribution of the four CERT categories in our patients, frequencies appeared to be roughly comparable between treated patients and untreated patients, and we did not find a statistically significant difference (Supplementary Figure S2). 

**Figure 2:**
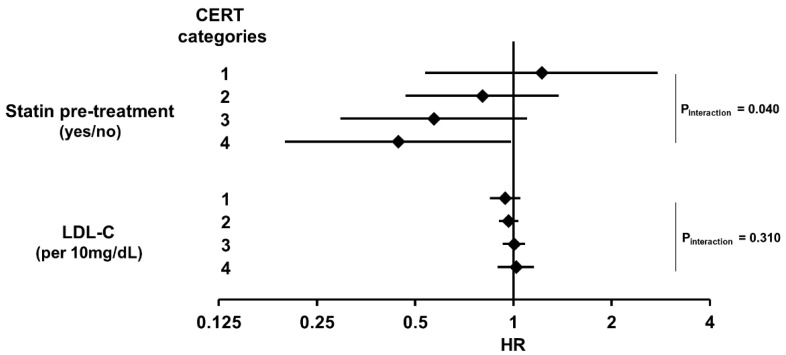
Association of statin treatment and LDL-C with overall mortality in PAD patients with respect to four CERT risk groups. The forest plot shows results from Cox proportional hazard regression analyses which are presented as hazard ratios and 95% confidence intervals.

Concerning the outcome, we did not find a significant effect of statin treatment before enrollment on the outcome of the total study population, but a trend demonstrating a 26% lower mortality risk under treatment was identified (HR = 0.742 [0.533–1.032], *p* ≤ 0.076). We then separated patients according to the four CERT categories. Applying this stratification, we found that the impact of statin treatment increased with increasing CERT categories (Figure 2) and was highest in category 4 (HR = 0.442 [0.200–0.987], *p* = 0.046). Moreover, the interaction between statin treatment and CERT categories was significant (*p* = 0.040), meaning that CERT categories had a significant impact on the association between statin treatment status and patients’ outcomes. By contrast, the LDL-C concentration at the baseline did not correlate with CERT (r = 0.035, *p* = 0.495) and was not associated with patients’ outcomes in the total cohort (HR per 10 mg/dL increase = 0.976 [0.934–1.019], *p* = 0.270) or in any of the subgroups (Figure 2) and there was also no significant interaction between the CERT categories and LDL-C (*p* = 0.310).

## 4. Discussion

### 4.1. Main Findings

From our data, we can conclude that in PAD patients, the ceramide-based risk score CERT is a significant and powerful predictor for 10-year overall survival as well as for cardiovascular survival and MACE. This predictive power was not observed with LDL-C. Regarding the effect of statins on patients’ outcomes, we also found that the categorization of the PAD patients according to CERT had a significant impact: the protective effect of statin treatment was more pronounced with increasing CERT risk categories. 

### 4.2. Ceramides as Risk Predictors

In this study, we used the CERT score to assign 379 PAD patients to one of four risk categories. This categorization, according to CERT, significantly predicted overall mortality as well as cardiovascular mortality and MACE, which is in line with previous data reporting the use of CERT in patients with CAD [17,22]. Considering the clinical significance of ceramides in predicting cardiovascular risk, there is a pertinent inquiry regarding their molecular characteristics. Ceramides are bioactive lipids [9] and mediate the buildup of signal platforms based on lipid rafts [23]. They also regulate cellular functions via the activation of protein phosphatases of the PP1 and PP2A family [24] and downstream targets, including the serine/threonine protein kinase Akt [10]. This impact on signaling pathways could explain, in part, their role described for metabolism and their predictive power regarding the development or prevention of disease [10]. Given their role (i) as a kind of a hub in lipid metabolism [9], (ii) in the uptake of lipids into the endothelial cell, (iii) in mediating lipotoxic events [10,25], and (iv) in promoting arterial dysfunction [26], they have recently been described as important and valuable predictors for increased cardiovascular event risk [11,26,27]. This study adds further color to the picture, demonstrating the value of ceramides, in particular, through the ceramide-based risk score CERT, as predictors of cardiovascular and overall risk in PAD patients. 

When making clinical decisions, the stratification of the baseline risk is suggested to be most applicable [28]. In our study, the power of statin treatment was stronger, with higher CERT categories reflecting a higher cardiovascular risk. This is in line with the well-known principle that the statin-mediated relative risk reduction for fatal endpoints is greater in people with a higher risk. The absolute benefits of statins are different concerning cardiovascular risk. This is true in primary prevention and also accounts for secondary prevention populations with previous cardiovascular disease events in which the benefit-to-harm balance of treatment is more favorable compared to individuals who have a lower average risk of cardiovascular disease [28,29]. Furthermore, in terms of cost-effectiveness, a previous study evaluated the health-economic justification of statin treatments in patients with borderline cardiovascular risk and found that statin therapy was more cost-effective in individuals with a higher risk of cardiovascular disease [30].

That said, a high cardiovascular risk may not necessarily be linked with a high LDL-C concentration and, thus, an existing high cardiovascular risk could be masked and treatment may be done less thoroughly or even fail to be initiated [31,32]. In contrast to ceramides, LDL-C was no significant predictor in our study, and there was no correlation between LDL-C and the CERT risk score. This was also true for ABI. In terms of LDL-C and its predictive value, current evidence increasingly highlights that within populations of cardiovascular disease patients, factors such as age, comorbidities, and lipid-lowering treatment compromise LDL-C-based risk prediction and alternative risk predictors might, thus, be more valuable [32,33]. Ceramides and ceramide-based risk scores, including CERT, are novel kinds of lipid biomarkers that are already deemed to outperform risk prediction by LDL-C [34] and are even particularly effective in discriminating PAD from CAD [8]. 

As the cardiovascular risk of PAD is higher than that of CAD, we suggest that statin treatment should be mandatory for PAD patients characterized to be in a high CERT category. 

However, our study shows a statistical interaction between statins and ceramides but cannot necessarily link biological statin action to ceramide function. In contrast to LDL-C, it is known that statins do not directly block ceramide synthesis, though there is a certain indirect statin effect on ceramide levels [35]. In our study, we did not see a clear and significant difference between statin-naïve patients and those under therapy concerning the ceramide levels represented by CERT categories. 

### 4.3. Ceramides as Treatment Targets 

The levels of ceramides in the bloodstream can be influenced by various factors, including lifestyle changes, like dietary modifications and regular exercise, as well as certain medications. 

In the context of the impact of diet, it is estimated that Western diets contain 200 to 400 mg of sphingolipids per day, with the majority being sphingomyelin, which is further broken down into ceramide [36]. It is, however, not yet clear how much dietary ceramides per se contribute to the body’s overall ceramide levels or how effectively they are absorbed or secreted after digestion. Nevertheless, it has been demonstrated that increased saturated fat intake in overweight subjects is capable of elevating the level of circulating ceramides in plasma [37]. Conversely, a Mediterranean diet may mitigate the potential deleterious effects of elevated plasma ceramide concentrations on CVD [38]. For instance, an 8-week fruit and vegetable intervention in young adults caused a decline in ceramide concentrations, along with improvements in their overall metabolic health and inflammatory status [39]. Though certain dietary interventions may impact circulating ceramide levels, further research with larger sample sizes is required to gain a more comprehensive understanding of the effects that specific nutrients have on the concentrations of specific ceramides. 

Apart from intake by diet, ceramides are also synthesized by de novo or salvage pathways, specifically at the endoplasmic reticulum’s surface. This process involves the combination of serine and palmitoyl-CoA, resulting in the formation of a ceramide precursor that possesses an additional fatty acyl group [40,41]. Once generated, ceramides are part of the eucaryotic cell membrane but they are also present in circulating lipoprotein particles, such as LDL-C. Hence, drugs like statins, ezetimibe, and PCSK9 inhibitors that are aimed at reducing cholesterol levels have the potential to lower serum ceramides as well [12,13,42]. As mentioned above, statins do not directly inhibit ceramide synthesis, but at least have a limited indirect effect [35]. In our study, we found slightly but not significantly lower CERT levels in treated compared to untreated patients. On the other hand, it has already been shown that inhibiting PCSK-9, which interferes with LDL-receptor degradation, reduces not only LDL-C but also, to an even larger extent, ceramide concentrations [13]. This implies that there might be a connection between the action of PCSK-9, the up-regulation of LDL receptors, and the levels of some circulating ceramides. Such a relationship could potentially contribute to cardiovascular risk reduction through the use of PCSK-9 inhibitors. Moreover, an indirect reduction in ceramide concentration might also be achieved using ezetimibe [43,44]. However, both these drug classes had no impact on our study results since the recruitment of our patients took place between 2006 and 2010 when ezetimibe was not broadly prescribed in Austria and PCSK-9 inhibitors were not even approved for prescription. Apart from this, fibrates, which are used to manage lipid levels, are an additional drug class that is reported to be associated with significant reductions in ceramide concentrations [45]. However, given the poor role fibrates have demonstrated in reducing cardiovascular risk [46], it seems questionable whether fibrates might become useful as a ceramide-modulating drug class for lowering CVD risk. 

On the other hand, drugs that directly target ceramide levels are currently being developed, and this is based on the primary objective that atherosclerosis development can indeed be reduced by inhibiting de novo ceramide biosynthesis [47]. Thus, we strongly believe that the clinical significance of ceramides may gain further attention, though the role of ceramide as a target for reducing the risk of atherosclerotic disease, in particular, treating PAD patients, remains to be investigated in more detail.

### 4.4. Summary of Clinical Consequences 

These findings hold significant clinical implications. They highlight CERT as a promising approach for predicting overall mortality and cardiovascular outcomes in PAD patients. Notably, there are morphological differences between lower limb and coronary arteries, with a considerably higher prevalence of calcification in the lower limbs. Additionally, disparities in plaque morphology exist between PAD and CAD [48]. This might partially account for the heightened risk of PAD compared to CAD. Therefore, effective medical intervention with statins, offering cholesterol reduction, anti-inflammatory properties, and the stabilization of plaque, becomes even more crucial in PAD cases. This study strongly emphasizes that the positive impact of statin treatment is further magnified in patients at higher risk, as indicated by CERT.

### 4.5. Strengths and Limitations

The present study has strengths and limitations. A particular strength of this study is the high follow-up rate. Additional strengths are that the study comprises a very well-characterized patient cohort, that all patients were in a stable stage, and that PAD was sonographically proven. One limitation is the fact that we selected European patients with PAD, which consequently does not reflect the general population. The mean annual death rate among our study patients was 4.7%, which is about four times higher than in the general population of the EU (1.2%) [49]. Despite these limitations, it is important to emphasize that PAD patients are a high-risk population and require special clinical attention. Another limitation of our study is its observational design, which precludes any conclusions about the causal relationships between ceramides and the observed outcomes. Additionally, our analyses were based on single measurements and did not consider changes in parameters over time, which could have impacted the observed outcomes. We did not have access to data on patient adherence to medical treatment, which could also have influenced the outcomes. We also had no data regarding the genetic background of our patients, in particular concerning ceramides. Furthermore, we want to mention that we have only analyzed a limited set of ceramides previously described in the literature to be associated with cardiovascular risk. There are many more species whose roles remain to be analyzed. Finally, the underlying pathophysiological mechanisms linking ceramides with the outcome, as well as the different environmental factors on circulating ceramide concentrations, require further investigation.

## 5. Conclusions

In summary, our findings demonstrate that CERT serves as a robust predictor of the 10-year prognosis of patients with PAD beyond conventional clinical risk factors. These results support the established link between ceramides and adverse outcomes in high-risk patients. The use of ceramides, specifically CERT, as a tool for risk assessment and decision making represents a promising avenue for future research. 

## Figures and Tables

**Table 1:** Baseline characteristics of the total study cohort after stratification according to CERT categories.

	Total Cohort (n = 379)	Category 1 (Low Risk, n = 116)	Category 2 (Moderate Risk, n = 140)	Category 3 (High Risk, n = 77)	Category 4 (Very High Risk, n = 46)	*p*-Value
Age, years	67 [60–74]	65 [57–74]	67 [60–74]	70 [64–75]	71 [62–76]	0.007
Male sex, % (n)	72 (273)	73 (85)	75 (105)	73 (56)	59 (27)	0.127
Current smokers, % (n)	31 (89)	34 (24)	29 (32)	30 (20)	33 (13)	0.793
Ever smokers, % (n)	84 (317)	82 (95)	85 (119)	84 (65)	83 (38)	0.815
Body mass index, kg/m^2^	26 [24–30]	26 [24–30]	26 [24–30]	27 [24–30]	26 [23–31]	0.670
Creatinine, mg/dL	0.90 [0.77–1.05]	0.90 [0.79–1.00]	0.87 [0.77–1.00]	0.90 [0.70–1.15]	0.95 [0.72–1.26]	0.166
Hypertension, % (n)	94 (356)	93 (108)	97 (136)	88 (68)	96 (44)	0.698
T2DM, % (n)	40 (150)	38 (44)	34 (47)	43 (33)	57 (26)	0.033
CKD % (n)	20 (75)	16 (18)	15 (21)	25 (19)	37 (17)	0.001
LDL-cholesterol, mg/dL	102 [81–125]	96 [83–119]	105 [81–126]	111 [79–149]	92 [76–114]	0.716
Statin use at baseline, % (n)	70 (266)	78 (91)	66 (92)	62 (48)	76 (35)	0.247
Rutherford score	2 [1–3]	2 [1–3]	1 [0–3]	3 [1–3]	3 [1–4]	0.006
Ankle–brachial index	1.0 [0.8–1.2]	1.0 [0.9–1.2]	1.0 [0.9–1.2]	1.0 [0.8–1.1]	0.9 [0.7–1.1]	0.047

**Table 2:** Association of CERT with study endpoints.

		Overall Mortality		Cardiovascular Mortality		MACE	
		HR [IQR]	*p*	HR [IQR]	*p*	HR [IQR]	*p*
CERT categories (4 steps)	Model 1	1.351 [1.157–1.578]	<0.001	1.427 [1.123–1.812]	0.004	1.255 [1.062–1.482]	0.008
	Model 2	1.259 [1.074–1.476]	0.005	1.349 [1.055–1.726]	0.017	1.225 [1.033–1.452]	0.020
	Model 3	1.265 [1.079–1.484]	0.004	1.360 [1.062–1.741]	0.015	1.221 [1.026–1.452]	0.024
	Model 4	1.215 [1.035–1.427]	0.017	1.304 [1.018–1.670]	0.035	1.167 [0.981–1.388]	0.081
CERT (13 steps)	Model 1	1.089 [1.040–1.140]	<0.001	1.124 [1.046–1.207]	0.001	1.062 [1.010–1.117]	0.019
	Model 2	1.067 [1.019–1.118]	0.006	1.105 [1.027–1.188]	0.007	1.065 [1.013–1.119]	0.014
	Model 3	1.068 [1.019–1.120]	0.006	1.105 [1.027–1.188]	0.008	1.062 [1.010–1.117]	0.019
	Model 4	1.055 [1.007–1.106]	0.024	1.090 [1.014–1.172]	0.020	1.049 [0.998–1.103]	0.061

Hazard ratios (HR) with the interquartile range (IQR) of Cox regressions are summarized applying different adjustment models. Model 1 represents an unadjusted regression. Model 2 contains age and sex. Model 3 contains age, sex, BMI, and LDL-C. Model 4 contains age, sex, BMI, LDL-C, T2DM status, hypertension status, CKD status, ever-smoking status, and statin treatment status. The HR is given either per 1 category increase in the categorized CERT score (4 categories = 4 steps) or per 1 step increase in the CERT score comprising 13 steps.

## Data Availability

The data that support the findings of this study are available from the corresponding author upon reasonable request.

## Supplementary Materials

The following supporting information can be downloaded at: https://www.mdpi.com/article/10.3390/jcm12196151/s1, Figure S1: Cardiovascular mortality and MACE in PAD Patients with Respect to CERT risk groups; Figure S2: Frequency of CERT risk categories in patients with respect to their pre-baseline treatment status with statins; Table S1: Lipids and their respective ions used in the study; Table S2: Hazard ratios of single ceramides for the primary study endpoint overall mortality.

## Abbreviations

CAD	Coronary artery disease
CERT	Coronary event risk test
CKD	Chronic kidney disease
CVD	Cardiovascular disease
HR	Hazard ratio
LC-MS/MS	Liquid chromatography-tandem mass spectrometry
LDL	Low-density lipoprotein
MACE	Major adverse cardiovascular events
T2DM	Type 2 diabetes mellitus
PAD	Peripheral artery disease
