# Supplementary material for: Coronary Event Risk Test (CERT) as a Risk Predictor for the 10-Year Clinical Outcome of Patients with Peripheral Artery Disease
Source: J Clin Med. 2023 Sep 23;12(19):6151. doi: 10.3390/jcm12196151 (PMC10573503; doi:10.3390/jcm12196151)
Supplement: Supplementary file 1 [file jcm-12-06151-s001.zip › jcm-2417887-supplementary.pdf]

Supplemental Data

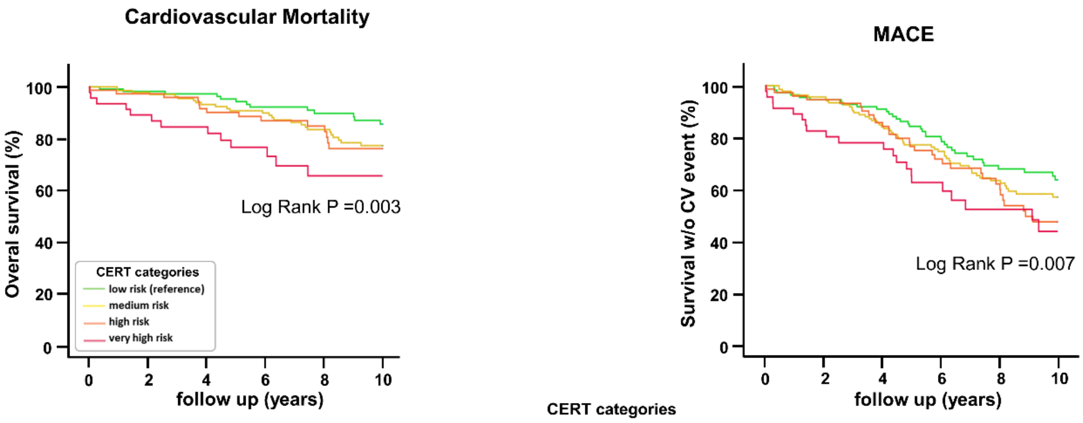

CERT categories						
1 (low risk)	112	106	100	86	70	58
2 (moderate ris k)	138	130	120	102	84	65
3 (high risk)	77	70	63	51	39	34
4 (very high risk)	46	40	34	25	17	15

CERT categories						
1 (low risk)	112	105	98	81	54	44
2 (moderate risk)	138	129	110	86	62	47
3 (high risk)	77	68	58	42	30	23
4 (very high risk)	46	38	32	22	14	10

Supplementary Figure S1. Cardiovascular mortality and MACE in PAD Patients with Respect to CERT risk groups. Kaplan-Meier estimates of cumulative probabilities of cardiovascular mortality and MACE are depicted according to the four risk groups of CERT. The number of patients at risk is given for each risk group every two years.

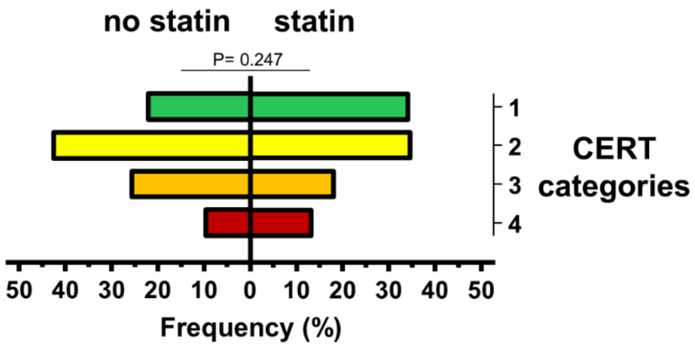

Supplementary Figure S2. Frequency of CERT risk categories in patients with respect to their pre-baseline treatment status with statins. P-value was calculated according to the Mantel-Haenszel test of trend.

Supplementary Table S1.

Ceramides	Q1 (M+HCOO)- (m/z)	Q1 (M+HCOO)- (m/z)	Collision energy	Internal standard
18:1/16:0	538.5	264.25	40	18:1/16:0 – d4
18:1/18:0	566.5	264.25	40	18:1/18:0 – d4
18:1/24:0	650.6	264.25	40	18:1/24:0 – d4
18:1/24:1	648.6	264.25	40	18:1/24:1 – d4

Lipids and their respective ions used in the study.

Supplementary Table S2.

Ceramides	HR	CI	P
18:1/16:0	1.189	1.034-1.368	0.015
18:1/18:0	1.112	0.955-1.296	0.173
18:1/24:0	0.825	0.693-0.983	0.031
18:1/24:1	1.116	0.957-1.301	0.162

Hazard ratios of single ceramides for the primary study endpoint overall mortality. Hazard ratios (HRs) are given after z-transformation with the 95% confidence interval (CI) and the respective p-value (P).
